# Cocaine use disorder patients develop distinct patterns of regulation of acth secretion by a vasopressin agonist and oxytocin: Report on a laboratory study

**DOI:** 10.1016/j.dadr.2023.100158

**Published:** 2023-04-11

**Authors:** Wilfrid Noël Raby, Matthew Heller, Demetrios Milliaressis, C. Jean Choi, Cale Basaraba, Frances R. Levin, Sarah Church, Martina Pavlicova, Edward V. Nunes

**Affiliations:** a Division on Substance Abuse, Department of Psychiatry, Montefiore Medical Center, Albert Einstein College of Medicine, 1510 Waters Place, 2nd Floor, Bronx, NY, 10461; b Division of Mental Health Data Science, New York State Psychiatric Institute, New York, NY, USA; c Department of Biostatistics, Mailman School of Public Health, Columbia University, New York, NY, USA; d Division on Substance Use Disorders, Department of Psychiatry, Columbia University Irving Medical Center and the New York State Psychiatric Institute, New York, NY, USA; e Wholeview Wellness Centers, 369 Lexington Avenue, Suite 14A, New York City, NY, 10017, USA

**Keywords:** Intranasal, Oxytocin, Cocaine, Stress, ACTH

## Abstract

**Background:**

***:*** Oxytocin and Vasopressin systems in the brain sustain adaptation to stressors. Cocaine being a stressor, it may alter brain homeostatic function. This dysregulation may entrench cocaine use disorder.

**Method:**

***:*** This is a human laboratory study of the effects of intranasal desmopressin (a Vasopressin 1b receptor agonist) and oxytocin on ACTH secretion in cocaine use disorder patients versus a control group. It consisted of two endocrine challenges performed on consecutive days. On day 1, the effect of intranasal desmopressin (80 IU) on ACTH secretion was measured. On day 2, a pre-treatment with intranasal oxytocin (24 IU) preceded intranasal desmopressin to monitor its effect on desmopressin-induced ACTH secretion. We hypothesized that the effect of intranasal oxytocin in controls would differ from the effect in cocaine use disorder patients.

**Results:**

***:*** Forty-three patients were included in this study: 14 controls and 29 cocaine use disorder patients. Significant differences were noted in the direction of change of ACTH secretion between the two groups. In cocaine use disorder patients, overall ACTH secretion was on average 2.7 pg/ml/min higher after intranasal desmopressin than after intranasal oxytocin/desmopressin (t_292_ = 2.91, *p* = 0.004). The opposite was observed in controls: overall ACTH secretion averaged 3.3 pg/ml/min less after intranasal desmopressin than after intranasal oxytocin/desmopressin (t_292_ = -2.35, *p* = 0.02).

**Conclusion:**

***:*** Intranasal oxytocin and desmopressin revealed a pattern of ACTH secretion in cocaine use disorder patients that is distinct from a non-addicted control group. (ClinicalTrial.gov00255357, 10/2014)

## Highlights

• Cocaine use disorder patients display a different regulation of ACTH secretion than a control sample in this human laboratory study.
• Intranasal Desmopressin increased ACTH secretion more than after a similar procedure preceded by a treatment with intranasal Oxytocin for cocaine use disorder patients.
• Intranasal Desmopressin decreased ACTH secretion more than after a similar procedure preceded by a treatment with intranasal Oxytocin for the control sample.

## 1 Introduction

Adaptive systems that regulate the impact of stress are important for survival, sustaining us in illness, during physical or mental exertion, and in the face of physiological challenges such as those imposed by drug addiction (Koob et al., 2014). Drugs of abuse are supra-natural stimuli that alter adaptation systems at the genetic, molecular and physiological level, to the point of being non-functional without the drug itself (Raby et al., 2008). These changes entrench addiction physiologically and psychologically (Koob and Kreek, 2007).

Quotidian stressors, with or without drug cues, can cause relapse to cocaine and other drugs (Preston et al., 2018). Sinha and colleagues (Sinha et al., 2006) demonstrated that stress can induce cravings for cocaine that predict relapse, and that these cravings parallel alterations in the Hypothalamo-Pituitary-Adrenal axis (HPA) and its hormonal cascade involving Corticotropin Releasing Factor (CRF), Adrenocorticotropin (ACTH), and Cortisol. The HPA axis is regulated by numerous neurotransmitters including neuropeptides such as oxytocin and vasopressin (Lee & Weerts, 2016). Under conditions of chronic stress, vasopressin sustains this vital hormonal cascade by being co-secreted with CRF to stimulate ACTH secretion (Gilles et al., 1982; DeGoeij et al., 1991; Aguilarae 1994, Tanoue et al., 2004; Zhou et al., 2011). In the main, oxytocin exerts a restraining effect on ACTH secretion (Dabrowska et al., 2011); chronic stress conditions alter the oxytocin neural system to diminish this regulation (Liberzon and Young, 1997; Kovacs et al., 1998).

Recurrent alcohol or cocaine use was reported to produce chronic stress effects, depleting oxytocin stores in the hypothalamus and amygdala (Sharnyai et al., 1992; Suvakina et al., 2006), and increasing oxytocin receptors at these sites (Liberzon and Young, 1997). Consequently, cocaine abuse may create a hormonal environment sensitive to exogenous oxytocin. This sensitivity may be reflected in the pattern of ACTH secretion, which may serve as a window on extrahypothalamic stress regulation (Windle et al., 2004; Martocchia et al., 2011). As oxytocin and vasopressin (and its synthetic V1b receptor agonist desmopressin) are small polypeptides or molecules, these can be administered by an intranasal route to deliver them at extra- and intra-hypothalamic sites (Buijs, 1978; Tribollet,^1992^; Banks, 2008), where some of the pathology of cocaine use disorder is thought to arise (Lee et al., 2020).

We present in this paper the results of a human laboratory study investigating the patterns of ACTH secretion in response to a challenge with intranasal desmopressin and to another challenge in which intranasal desmopressin insufflation is preceded by a pre-treatment with intranasal oxytocin. In accordance with the findings that chronic stress increases the effect of vasopressin on ACTH secretion, we hypothesized: 1) Intranasal desmopressin would increase ACTH secretion in cocaine use disorder patients more than in a non-addicted control group; 2) Pre-treatment with intranasal oxytocin would blunt the ACTH secretion induced by intranasal desmopressin more so in the cocaine use disorder group than in a non-addicted control group. The primary outcome was participant ACTH levels. This study was conducted in parallel with a double-blind, randomized, placebo-controlled 6-week clinical trial of intranasal oxytocin (24 IU). Results from this study are already published (Raby et al., 2022). It revealed that intranasal oxytocin increased the odds of abstinence 15:1 compared to placebo at week 6. The laboratory study sought a mechanistic understanding of this effect of intranasal oxytocin on the odds of abstinence in cocaine use disorder.

## 2 Material and methods

### 2.1 Study participants

**Fig. 1:**
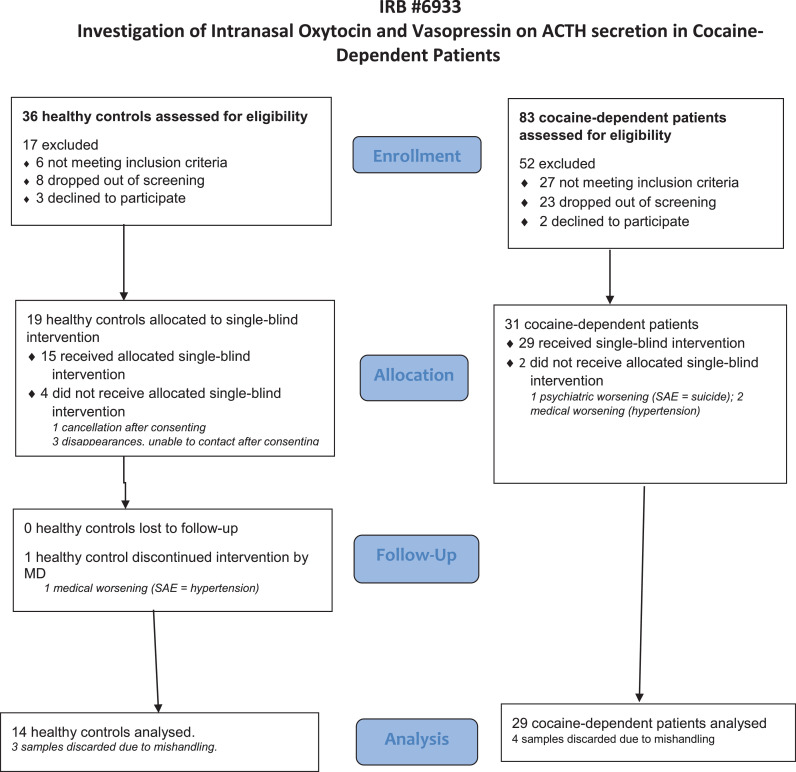
Consort Statement.

Participants were recruited by local advertising. The study was conducted at the STARS clinic, a research clinic in the Division on Substance Use at the New York State Psychiatric Institute (NYSPI) and Columbia University. It was approved by the NYSPI Institutional Review Board (IRB 6093) and all participants gave a written informed consent. Fig. 1 presents the Consort diagram.

Medical screening included a history and physical examination, urine toxicology to verify cocaine use in cocaine use disorder patients, pregnancy test for women, an electrocardiogram, a comprehensive metabolic panel, and complete blood count. We used the MINI International Neuropsychiatric Interview to confirm or refute the diagnosis of cocaine use disorder or of comorbid psychiatric disorder in cocaine patients and controls (Lecrubier et al., 1997). To verify the integrity of the olfactory systems in intranasal cocaine users, they were administered the Picture Smell Identification test (PSIT, Suzuki et al., 2004). All patients and controls passed the PSIT. Study inclusion criteria required that cocaine use disorder patients were: 1) between the age of 18–60; 2) met DSM-IV criteria for current cocaine dependence and that they were seeking treatment; 3) displayed at least one cocaine-positive urine toxicology during screening; 4) used cocaine weekly, at least 4 days in the past 30 days; 5) no current hyponatremia (Na ≤ 135 meq/L, exclusion criteria); 6) for women, a negative pregnancy test and written assurance of the use of a contraceptive during the study. Study inclusion criteria for controls were: 1) no substance use disorder by history or being in remission for the past 2 years; 2) no active medical illness; 3) no psychiatric history; 4) absence of hyponatremia; 5) for women, a negative pregnancy test and written assurance of the use of a contraceptive during the study. The study was conducted from December 2014 to May 2016.

### 2.2 Study design

This study consisted of two intranasal endocrine challenges administered on 2 separate but consecutive days (Table 2). For cocaine use disorder patients, these took place during a 7-day inpatient abstinence induction period that preceded the clinical trial (Raby et., 2022). Patients and controls had to produce a negative urine toxicology test before the challenge procedures and had not had any food or drink for at least 2 h before the challenges. The challenges were conducted at approximately 10 AM on consecutive days. After an acclimation period (60 min) following the insertion of an intravenous catheter, baseline measurements of ACTH levels in serum samples were collected at 30, 20, & 10 min before each challenge. On Day 1, after the baseline period, patients and controls were challenged with an intranasal insufflation of 80 IU of desmopressin and ACTH levels were measured at 10, 20, 30, 45, 90 min afterwards. Desmopressin was chosen for its low affinity to oxytocin receptors, its greater selectivity for the V1b receptor compared to vasopressin, and for its longer half-life than vasopressin itself. Thus, desmopressin could be more revealing of our hypothesis concerning the role of V1b receptors in the adaptation of the HPA axis to cocaine use disorder (Manning et al., 2012).

On Day 2, patients and controls were given first a pre-treatment with 24 IU of intranasal oxytocin before receiving the same intranasal desmopressin challenge. Measurements of ACTH levels in serum samples were collected at 10, 20, 30, 45, and 90 min after the desmopressin challenge, as well as 10, 20, 30 min after the oxytocin pre-treatment.

After collection, serum samples were frozen at −20 °Celsius, packed on dry ice, and shipped to Nathan Kline Laboratory in Orangeburg NY for analysis by ELISA methodology (www.enzolifesciences.com). Intra- and inter-coefficient of variability for this analysis were 3 and 7% respectively.

### 2.3 Training for intranasal administration

All cocaine use disorder patients and controls were trained by staff to self-administer intranasal solutions for the laboratory sessions (Born et al., 2002; Dhuria et al., 2010). Solutions of desmopressin (80 IU, Pfizer USA) or oxytocin (24 IU, Novartis of Switzerland) were drawn by a research pharmacist into pre-prepared syringes adapted with an intranasal insufflator. At the time indicated during the laboratory protocol (Table 2), patients and controls would self-administer the solutions with the prepared syringes under supervision of research staff.

### 2.4 Compensation

For their participation in the study, each control and cocaine use disorder patients received $150. We covered transportation costs ($5).

### 2.5 Data analysis

ACTH secretion outcomes were analyzed using a longitudinal mixed effects model with a random intercept to account for between-subject variability and a random effect of time from baseline to account for within-subject repeated measures. The fixed effects of baseline ACTH, patient group, time, and endocrine challenge was included in the model. The 3-way interaction of group*time*challenge was tested to examine the primary hypothesis of whether ACTH secretion provoked by intranasal desmopressin or intranasal oxytocin/desmopressin differs between cocaine and control patients, while adjusting for their baseline ACTH secretion. If the 3-way interaction was not significant, it was removed from the model, as well as any non-significant 2-way interactions between variables group, time, and challenge in the model.

Table 1 shows the timetable of the endocrine challenges and ACTH measurements. Subject-specific mean baseline ACTH secretion was computed as the mean of three measurements of ACTH during the baseline evaluation period (130, 140, 150 min).

**Table 1:** Timetable of Endocrine challenges and ACTH measurements.

	**Phase 1**	
**Time (min)**	**Day 1**	**Day 2**
	**Desmopressin Endocrine Challenge**	**Oxytocin/Desmopressin Endocrine Challenge**
**0**	Saline catheter inserted	
**30**	Stabilization and Adaptation period	
**60**		
**90**		
**120**		
	*Baseline Evaluation Period*	
**130**	ACTH	ACTH
**140**	ACTH	ACTH
**150**	ACTH	ACTH
**160**	IN Desmopressin (80 IU)	IN Oxytocin (24 IU)
	*Post-* Desmopressin *Challenge Period*	*Post-Oxytocin Pre-Treatment Period*
**170**	ACTH	ACTH
**180**	ACTH	ACTH
**190**	ACTH	ACTH
**200**		IN Desmopressin (80 IU) administered
		*Post-Oxytocin/Desmopressin Challenge Period*
**205**	ACTH	
**210**		ACTH
**220**		ACTH
**230**		ACTH
**245**		ACTH
**250**	ACTH	
**290**		ACTH

## 3 Results

### 3.1 Sample characteristics

Table 2 shows the demographic and clinical characteristics of the 43 participants in the study. Twenty-nine (67%) had cocaine use disorder and 14 belonged to the control group. The sample was predominantly African American (84% of total sample) and male with on average 13 years of education (SD = 2). Among the cocaine use disorder patients, the average age of first use of cocaine was 21 (SD = 6) and 25 for regular use (SD = 7). There was a significantly higher proportion of African American participants in the cocaine use disorder patient group (Fisher's Exact test *p* = 0.012).

**Table 2:** Demographic characteristics at baseline (*n* = 43).

	**Total Sample**		**Control**		**Cocaine-Patient**		
	**(*n*** **=** **43)**		**(*n*** **=** **14)**		**(*n*** **=** **29)**		
**Characteristic**	**n**	**Mean (SD)**	**n**	**Mean (SD)**	**n**	**Mean (SD)**	**p-value_1_**
		**or%**		**or%**		**or%**	
**Demographics**							
Age	43	47.7 (8.5)	14	43.0 (10.7)	29	50.0 (6.3)	.010
Sex							.210
Male	30	69.8%	8	57.1%	22	75.9%	
Female	13	30.2%	6	42.9%	7	24.1%	
Race							.012
African American	36	83.7%	9	64.3%	27	93.1%	
White	2	4.7%	2	14.3%	0	0.0%	
Asian	2	4.7%	2	14.3%	0	0.0%	
Hispanic/Latino	3	7.0%	1	7.1%	2	6.9%	
Years of Education	39	13.2 (1.9)	13	13.8 (2.1)	26	12.9 (1.7)	.140
Annual Income							.211
<$20K	6	14.0%	1	7.1%	5	17.2%	
$20K-$30K	7	16.3%	3	21.4%	4	13.8%	
>$30K	6	14.0%	4	28.6%	2	6.9%	
N/A	24	55.8%	6	42.9%	18	62.1%	
**Days of Cocaine Use for the 30 days prior to study entry**	–	–	–	–	29	11.2 (5.5)	
**Age of Substance Use**							
Age of 1st Cocaine Use	–	–	–	–	24	20.9 (5.5)	
Age of Regular Cocaine Use	–	–	–	–	17	25.1 (7.0)	
Age of 1st Crack Cocaine Use	–	–	–	–	24	27.5 (8.0)	
Age of Regular Crack Cocaine Use	–	–	–	–	23	28.7 (7.3)	
Age of 1st Marijuana Use	–	–	–	–	24	15.8 (3.5)	
Age of Regular Marijuana Use	–	–	–	–	11	16.3 (3.4)	
Age of 1st Alcohol Use	–	–	–	–	26	15.5 (3.4)	
Age of Regular Alcohol Use	–	–	–	–	18	18.6 (4.3)	
Age of 1st Nicotine Use	–	–	–	–	21	16.2 (2.6)	
Age of Regular Nicotine Use	–	–	–	–	19	17.3 (3.3)	

1Two-sample *t*-test, chi-squared test, or Fisher Exact test.

### 3.2 Endocrine challenge outcomes

**Fig. 2:**
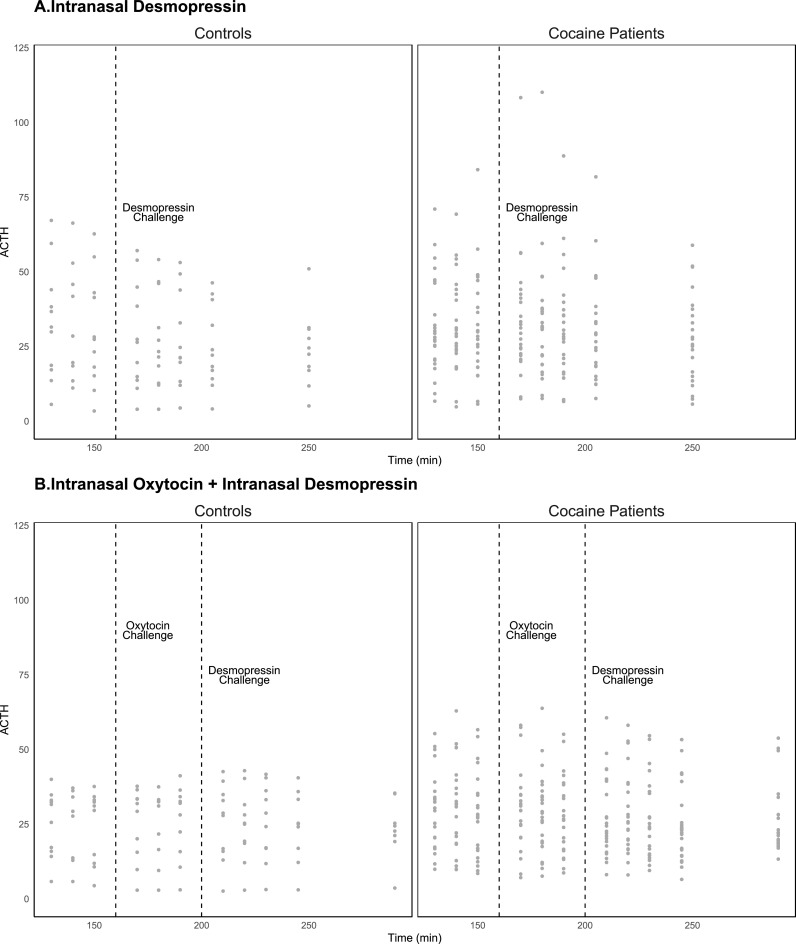
Observed ACTH Secretion for Control and Cocaine Patients by Intranasal Desmopressin and Intranasal Oxytocin + Intranasal Desmopressin challenges.

To simply describe the outcomes, mean and standard deviation baseline levels of ACTH were computed. For cocaine patients (*n* = 25), mean (SD), baseline ACTH levels was 32.4 (15.7) and 30.9 (18.9) for controls (*n* = 11), which were not significantly different between groups (t_34_ = −0.24, *p* = 0.81). Fig. 2 shows observed ACTH levels across groups and endocrine challenge type.

**Fig. 3:**
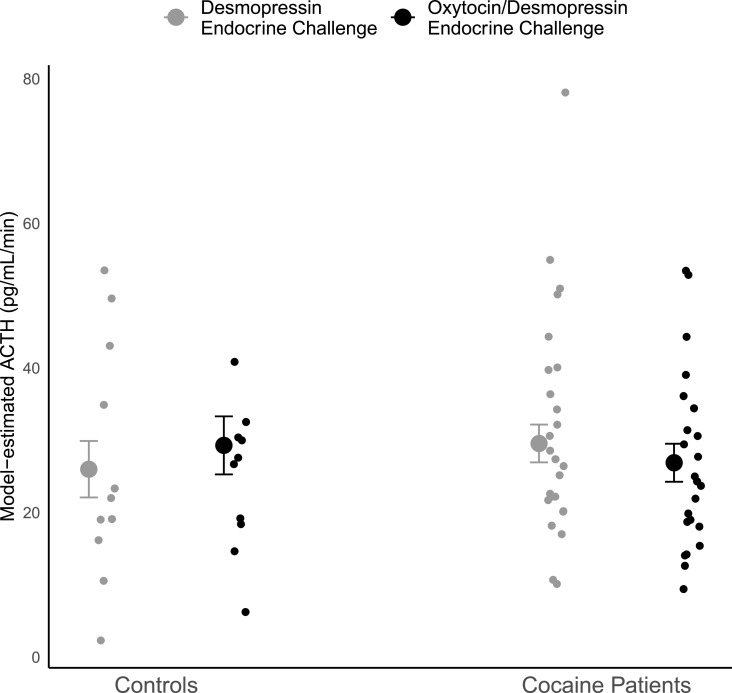
Model-estimated Mean ACTH Secretion with 95% confidence intervals for Controls and Cocaine Patients by Intranasal Desmopressin and Intranasal Oxytocin + Intranasal Desmopressin challenges, adjusted by baseline ACTH levels. Model-estimated ACTH Secretion for each participant are also presented. The Mean ACTH difference across challenges in Controls was 3.3 pg/ml/min (*p* = 0.02) and in Cocaine Patients was −2.7 pg/ml/min (*p* = 0.004).

The 3-way interaction of group*time*challenge was not significant (F_4,__280_ = 0.15, *p* = 0.97) suggesting no differences in ACTH secretion over time between endocrine challenge type and group. When the non-significant 3-way interaction was removed, there was a significant 2-way group*challenge interaction (F_1,__292_ = 13.0, *p* = 0004). Fig. 3 shows the significant differences in the direction of change in ACTH secretion, after adjusting for baseline ACTH: among cocaine use disorder patients, overall ACTH secretion was on average 2.7pg/ml/min higher after intranasal desmopressin than after intranasal oxytocin/desmopressin (t_292_ = 2.91, *p* = 0.004). The opposite was observed among controls: ACTH secretion averaged 3.3pg/ml/min lower after intranasal desmopressin than after intranasal oxytocin/vasopressin (t_292_ = −2.35, *p* = 0.02), while controlling for baseline ACTH levels. However, there was no significant difference in ACTH levels overall between patients and controls within either challenge. There were no gender differences between mean ACTH level changes related to the endocrine challenges.

## 4 Discussion

The results of this study point to a difference in the regulation of the HPA between cocaine use disorder patients and a non-addicted control group. Namely, among cocaine use disorder patients, desmopressin, likely acting at Vasopressin 1b receptors, increased ACTH secretion, which was significantly diminished by a pretreatment with oxytocin. The contrary was observed in a control sample: desmopressin alone had a lesser impact of ACTH secretion, a pretreatment with oxytocin increased ACTH secretion. These findings support our hypothesis that among those with cocaine use disorder, adaptations in stress regulation, as unveiled by measuring ACTH levels, involve vasopressin and oxytocin. Although the roles of vasopressin and oxytocin in the adaptation of the HPA axis to chronic stress is widely acknowledged (see Aguilara & Rabadan-Diehl, 2000), we know of no other study that has sought to verify this in humans with cocaine use disorder.

There is controversy about the use of desmopressin to study vasopressin-based adaptations in corticotrophs in the adenohypophysis. Aside from primary Cushing's disease caused by corticotropinomas, desmopressin has not been shown to elicit ACTH secretion under basal, non-neoplastic conditions (Luque et al., 2013). In Cushing's disease, it is speculated that the effect of desmopressin reflects an increased density of Vasopressin 1b receptors on the surface of corticotropinoma cells far beyond what is measured in normal corticotrophs (Luque et al., 2013). Hence, increased vasopressin 1b receptor density may occur either in pathological states or possibly under some or all chronic stress conditions. If this is so, the effect of desmopressin on ACTH secretion reported in this study suggests that cocaine use disorder, similar to Cushing's disease, may exist beyond the boundaries of basal constitutional conditions, in which the sensitivity of corticotrophs to vasopressin or desmopressin is low (Sakai et al., 1997).

As stated, the results observed in controls differed from those in cocaine use disorder patients: in controls, intranasal desmopressin did not significantly change ACTH levels from baseline, but the treatment with intranasal oxytocin preceding intranasal desmopressin led to an increase in ACTH secretion, suggesting a different receptor interaction between vasopressin and oxytocin on corticotrophs. Although the binding affinity data would imply little interaction of oxytocin at the human V1b receptor (Manning et al., 2012), other investigation looking at ACTH secretion suggest that such interaction may take place. Link et al. (1992) reported in rodent isolated adenohypophysis cells that oxytocin could exert a synergistic or additive effect to CRF or vasopressin-induced secretion. Another rodent study by Schlosser et al. (1994) reported that oxytocin can cause ACTH secretion by stimulation of Vasopressin 1b receptors. In healthy human controls, two studies have reported inhibitory influences of oxytocin on ACTH secretion (Legros, 1982; Legros et al., 1984), while another reported no effect (Lewis and Sherman, 1985). An explanation offered by investigators is that effects on stress from oxytocin may depend on the strength of emotional or stress-laden context (Guzman et al., 2013; Sharnay-Tsoory S & Abu-Akel A, S 2016). While our data cannot describe exactly the receptor mechanism by which our observed pattern of ACTH secretion was produced in our control sample, it does illustrate that cocaine use disorder patient develop differing ACTH response to oxytocin, most likely as a result of chronic cocaine use and associated psychosocial stressors operating as the driving stressor influencing the HPA axis.

Limitations to this study include the small sample size, and the differences between control and cocaine-disorder patient samples, and the fixed-order design, all of which could limit generalizability. As our cocaine-dependent patient sample, composed from the respondents to our advertising, was made up almost entirely of males and African American patients, genetic and gender differences could have produced a pattern of results that cannot be extended to all cocaine use disorder patients. Future research should first aim at corroboration of these results in larger samples of human cocaine-disorder patients with better matched control samples. Randomizing the order of the endocrine challenge may enhance the relevance of our results to all cocaine patients; we considered that this fixed order (intranasal desmopressin on day one, intranasal oxytocin pretreatment before intranasal desmopressin on day 2) would represent the usual circumstances of most cocaine patients in that repeated cocaine use occurs in absence of any therapeutic intervention.

As stated in the introduction, the laboratory study sought to understand how intranasal oxytocin could raise the odds of abstinence by testing the theory of chronic stress for its relevance to the treatment of cocaine use disorder. While each study unveiled significant effects, we could not find associations that tangibly linked the results of the laboratory and clinical trial studies. Hence, questions remain:: 1) Does either the desmopressin-induced increase in ACTH, or the decrease in ACTH due to intranasal oxytocin pretreatment relate to the increase in odds of abstinence from cocaine seen in the clinical trial? The lack of association may be because: 1) despite the voluminous literature on stress and addiction (see Koob et al., 2014), this aspect of the chronic stress theory does not apply; 2) ACTH levels may not be a relevant parameter. The difficulty in studying stress relates to its redundancy of purpose (such as fighting infection, sustaining physical and mental exertion) and the similar molecular vectors that sustain these many purposes (Selye, 1950); 3) limitations in this study (see above) may have depleted the statistical power to detect the aforementioned associations. Regrettably, the sought after associations must await potential validation in studies to come.

Some clinical trials that have yielded positive outcomes point to promising mechanisms. Suggestive of dysregulation in the dopaminergic pathways (Volkow et al., 2006) amphetamine-based clinical trials have yielded the most consistent positive outcomes (Castells et al., 2010; Herin et al. 2010; Rush and Stoop, 2012; Negus & Henningsfield, 2015) yet are reluctantly used due to concerns about diversion, although this risk may have been overstated among those seeking treatment for a cocaine use disorder (Levin et al., 2020). Similary, disulfiram, a medication capable of blocking the enzyme dopamine-beta-hydroxylase, has also shown benefit (Carroll et al., 1998), but is seldom used for fear that concurrent alcohol use would trigger a disulfiram reaction. Conventional glutaminergic medications, although promising (Zhou et al., 2014; Niedzielska-Andres et al., 2021), have not been effective (Bisaga et al., 2010), except for a report by Dakwar et al. (2019) in which IV ketamine coupled with mindfulness therapy improved abstinence from cocaine. However important this report is, the difficulty of widespread implementation of this approach remains. Targeting stress-related pathophysiology, guanfacine – a sympatholytic – has been reported to reduce relapse risk in cocaine disorder patients (Fox and Sinha, 2014), but remains underused. Hence the question that continues to confront researchers centers on which molecular, cellular, or physiologic lever can be pulled into an implementable treatment effective enough to warrant FDA approval that would sanction widespread use.

However preliminary, the results of this study and of our previously published clinical trial (Raby et al., 2022) imply that intranasal oxytocin may be an approach that could be pursued. Should the intricacies of its effects be the subject of continued research, and should its impact on abstinence be confirmed, intranasal oxytocin may represent a simple non-addictive approach to the treatment of cocaine use disorder that could be used in any front-line drug addiction clinic to support existing behavioral treatment.

## Funding source

This study was supported by a R21 award from the National Institute of Drug Abuse (NIDA) DA035461 and Supplement DA035461–02S1. NIDA did not have a role in the study design, collection, analysis, and interpretation of the data, nor in the writing of this manuscript or the decision by the authors to submit this report for publication.

## Contribution of authors

Dr. Wilfrid Noël Raby, PhD MD: Principal Investigator of this study. Dr. Raby conceptualized the study, applied for the R21 that funded the study, conducted data collection, supervised the integrity of the study and of the data collected, and wrote the manuscript. Matthew Heller, BSc MSc: Worked as a research assistant in the conduction of the study and reviewed all the collected data for accuracy. Demetrios Milliaressis, BSc, MSc, PhD (Honorary): Worked as a research assistant in the conduction, reviewed all the collected data for accuracy, and participated in the editing of the manuscript. Jean Choi MSc: Performed the data analysis for the study and participated in the final editing of the manuscript. Cale Basaraba, MPH: Participated significantly in the data analysis. Martina Pavlicova PhD: Oversaw the initial design of the study and the data analysis. Frances R. Levin MD: Provided mentorship and guidance to the PI, and participated in the editing of the final manuscript. Sarah Church PhD: Provided material support to the study. Edward V. Nunes MD: Provided mentorship and support during all phases of this study and participated in the editing of the final manuscript.

## Conflict of interest statement for authors

NIDA: Nothing to Declare. Dr. Wilfrid Noël Raby, PhD MD: Principal Investigator of this study. The PI has no conflict of interest to declare. Matthew Heller, BSc MSc: No conflict of interest to declare. Demetrios Milliaressis, BSc, MSc, PhD (Honorary): No conflict of interest to declare. Jean Choi MSc: No conflict of interest to declare. Cale Basaraba, MPH: No conflict of interest to declare. Martina Pavlicova PhD: No conflict of interest to declare. Frances R. Levin MD: No conflict of interest to declare. Sarah Church PhD: No conflict of interest to declare. Edward V. Nunes MD: No conflict of interest to declare.

## Author disclosures

NIDA: Nothing to Declare. Dr. Wilfrid Noël Raby, PhD MD: Principal Investigator of this study. Dr. Raby conceptualized the study, applied for the R21 that funded the study, conducted data collection, supervised the integrity of the study and of the data collected, and wrote the manuscript. The PI has no conflict of interest to declare. Matthew Heller, BSc MSc: Worked as a research assistant in the conduction of the study and reviewed all the collected data for accuracy. No conflict of interest to declare. Demetrios Milliaressis, BSc, MSc, PhD (Honorary): Worked as a research assistant in the conduction, reviewed all the collected data for accuracy, and participated in the editing of the manuscript. No conflict of interest to declare. Jean Choi MSc: Performed the data analysis for the study and participated in the final editing of the manuscript. No conflict of interest to declare. Cale Basaraba, MPH: Participated in the data analysis. No conflict of interest to declare. Martina Pavlicova PhD: Oversaw the initial design of the study and the data analysis. No conflict of interest to declare. Frances R. Levin MD: Provided mentorship and guidance to the PI, and participated in the editing of the final manuscript. No conflict of interest to declare. Sarah Church PhD: Provided material support to the study. No conflict of interest to declare. Edward V. Nunes MD: Provided mentorship and support during all phases of this study, and participated in the editing of the final manuscript.

## Declaration of Competing Interest

None of the authors hold any financial interests or partnerships with people or organizations that could bias the results or the writing of this manuscript.
